# Association between Surrogate Markers of Insulin Resistance and the Incidence of Colorectal Cancer in Korea: A Nationwide Population-Based Study

**DOI:** 10.3390/jcm13061628

**Published:** 2024-03-12

**Authors:** Minkook Son, Sang Yi Moon, Myeongseok Koh, Yeowool Kang, Jong Yoon Lee

**Affiliations:** 1 Department of Physiology, Dong-A University College of Medicine, Busan 49201, Republic of Korea; physionet@dau.ac.kr; 2 Department of Data Sciences Convergence, Dong-A University Interdisciplinary Program, Busan 49201, Republic of Korea; 3 Division of Gastroenterology, Department of Internal Medicine, Dong-A University College of Medicine, Busan 49201, Republic of Korea; sang4401@dau.ac.kr (S.Y.M.); kohdolchu@gmail.com (M.K.); kangyw56@gmail.com (Y.K.)

**Keywords:** insulin resistance, colorectal neoplasms, metabolic syndrome

## Abstract

**Background:** Insulin resistance (IR) is assessed using surrogate markers such as the triglyceride–glucose (TyG) index, the triglyceride-to-high-density lipoprotein cholesterol (TG/HDL-C) ratio, and the metabolic score for IR (METS-IR). Limited studies investigated their association with colorectal cancer (CRC) incidence, and no research has been conducted on their association with the METS-IR. **Method:** This study used claims data from the Korean National Health Insurance Service, analyzing a cohort of 314,141 Koreans aged over 40 who participated in the National Health Screening Program from 2009 to 2010. The follow-up period was extended until 31 December 2019. Participants were divided into four groups based on quartiles (Q1–Q4) of the markers. **Results:** All surrogate markers of IR had sequentially statistically lower disease-free probabilities from Q1 to Q4. The Cox proportional hazard model demonstrated statistically significant positive associations between CRC incidence and Q3 and Q4 of the TyG index, as well as Q3 and Q4 of the TG/HDL-C ratio and Q4 of the METS-IR. The constrained cubic spline method revealed a nonlinear, positive dose–response relationship between the TyG index and the METS-IR in relation to CRC incidence. **Conclusions:** In conclusion, the TyG index, TG/HDL-C ratio, and METS-IR were positively correlated with CRC incidence in Koreans.

## 1. Introduction

Colorectal cancer (CRC) is the third most common cancer and the third leading cause of cancer-related death worldwide, affecting both men and women [1]. Approximately 60–65% of incidences of CRC occur sporadically, independent of genetic factors, and can be attributed to modifiable environmental risk factors such as obesity, physical inactivity, an unhealthy diet, alcohol consumption, and smoking [2]. Metabolic syndrome, a condition strongly associated with these environmental factors, has been identified as a cluster of metabolic abnormalities linked to various types of malignancies, including CRC [3,4].

Insulin resistance (IR) is acknowledged as a crucial mechanism in the progression of metabolic syndrome and is closely associated with obesity, malignancy, non-alcoholic fatty liver disease, cardiovascular disease, polycystic ovary syndrome, and other abnormalities [5,6,7,8]. Recent findings suggest that IR is a contributing factor to the rise in obesity-related cancers, as reported by the International Agency for Research on Cancer and the Centers for Disease Control and Prevention, which have linked being overweight and obese to heightened risks of 13 different cancers [9,10]. IR is deemed significant in the pathogenesis of 13 cancer types, with a notable emphasis on CRC [11,12,13].

The hyperinsulinemic–euglycemic glucose clamp technique is considered the primary and most direct approach for quantifying insulin sensitivity and estimating IR. However, this approach is time-consuming, costly, labor-intensive, and technically complex [14]. The homeostatic model assessment of IR (HOMA-IR) serves as a mathematical model to assess IR indirectly [15]. However, its clinical utility is somewhat limited as it requires the measurement of fasting insulin, which is not routinely assessed unless the endocrine system is specifically evaluated. As there is an increasing trend toward the utilization of IR assessments in clinical settings, the importance of relatively simple markers has been emphasized. Therefore, clinicians require a convenient surrogate marker to assess IR in routine clinical practice. Previous research has shown that the triglyceride–glucose (TyG) index is closely associated with IR and serves as a marker of IR [16]. Another surrogate marker is the triglyceride-to-high-density lipoprotein cholesterol (TG/HDL-C) ratio, a simple indicator of IR that is strongly associated with HOMA-IR [17,18]. The most recently proposed measure of IR is the metabolic score for IR (METS-IR), which has been used as a new surrogate marker for IR because of its association with the development of type 2 diabetes mellitus, cardiovascular disease, dyslipidemia, and hyperuricemia [19,20,21,22].

Because of the challenges associated with direct measurements of IR, which is considered a key process in the development of CRC, surrogate markers, such as the TyG index, TG/HDL-C ratio, and METS-IR, have been proposed. However, only a few studies have investigated their association with the incidence of CRC [23,24,25]. Furthermore, no studies have investigated the association between METS-IR and CRC incidence. Therefore, the objective of this large cohort study was to assess the association between surrogate markers of IR and the incidence of CRC in specific subgroups, including sex, age, and obesity status, and to determine whether colon or rectal cancer was present in Korean individuals who participated in the National Health Screening Program (NHSP).

## 2. Materials and Methods

### 2.1. Study Design and Population

This retrospective population-based cohort study used claims data obtained from the National Health Insurance Service—National Health Screening Cohort (NHIS-HealS) in Korea. The NHIS is a single government insurer that provides a mandatory universal insurance system for the Korean population through which a database was established encompassing population statistics, treatment claims for hospitalizations and outpatient care, prescription drugs, medical procedures, and diagnoses according to the International Classification of Diseases, 10th edition (ICD-10). Additionally, the National Health Screening Program (NHSP) is provided to all insured individuals aged over 40 years every two years. This program includes a self-report questionnaire that collects sociodemographic data, anthropometric measurements, lifestyle behaviors, and laboratory test results. The NHIS-HealS database was constructed using data from NHSP participants.

**Figure 1:**
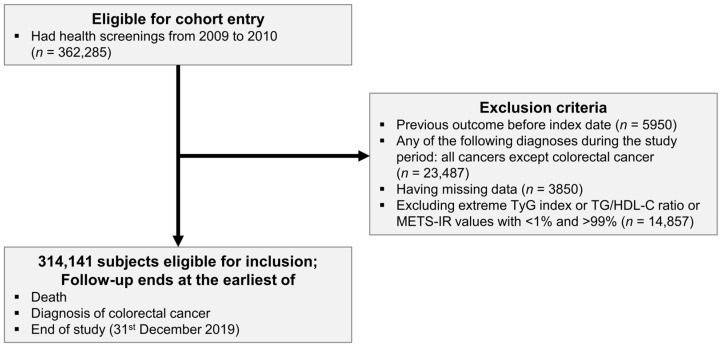
The flow of the selection of the study population.

We analyzed a cohort of 362,285 Korean adults aged over 40 years who participated in the NHSP from 2009 to 2010 by utilizing data from the NHIS-HealS database. From this cohort, we excluded 5950 individuals with CRC before the index date and 23,487 individuals who were diagnosed with cancers other than CRC during the follow-up period. Furthermore, we excluded 3850 individuals with missing data and 14,857 individuals with TyG index, TG/HDL-C ratio, or METS-IR values below the 1st or above the 99th percentile. Therefore, 314,141 individuals were included in the analysis. The follow-up period was extended until 31 December 2019 and ended earlier in the event of death or CRC diagnosis (Figure 1).

### 2.2. Definitions of Key Variables

The equations used to quantify IR, including the TyG index, TG/HDL-C ratio, and METS-IR, were evaluated based on levels of triglyceride (TG), fasting blood glucose (FBG), and high-density lipoprotein cholesterol (HDL-C) and the body mass index (BMI). The TyG index was calculated by taking a natural logarithm (ln) using the following equation: ln [TG (mg/dL) × FBG (mg/dL)/2]. The TG/HDL-C ratio was calculated using the following equation: TG (mg/dL)/HDL-C (mg/dL). The METS-IR was calculated using the following equation: ln [(2 × FBG (mg/dL) + serum TG level (mg/dL)] × BMI (kg/m^2^)/ln [serum HDL-C level (mg/dL)]. Participants were divided into four groups based on quartiles (Q1, Q2, Q3, and Q4) of the TyG index, TG/HDL-C ratio, and METS-IR. To identify individuals with newly diagnosed CRC for data analysis, we utilized NHIS data with a primary focus on insurance claims. Patients with CRC were identified based on the ICD-10 diagnostic codes C18, C19, and C20.

### 2.3. Definitions of Covariates

The Charlson comorbidity index (CCI) was computed using comorbidities [26]. To calculate the BMI, the body weight in kilograms was divided by the square of the height in meters (kg/m^2^). As per guidelines provided by the World Health Organization considering the Asia–Pacific perspective, obesity was defined as a BMI of ≥25 kg/m^2^. The laboratory results included hemoglobin levels and glomerular filtration rate (GFR) measurements. The self-report questionnaire collected data on current smoking, alcohol consumption, and regular exercise habits. Income level was classified into four quantiles, and residence was categorized as either urban or rural. Definitions of hypertension, diabetes, and dyslipidemia are provided in Supplementary Table S1.

### 2.4. Statistics and Data Analysis

Variables were reported as means with standard deviations or frequencies with percentages. We present a Kaplan–Meier curve to visualize the relationship between the surrogate markers of IR and CRC. To evaluate the risk of CRC, we used a Cox proportional hazards model with adjustments for multiple covariates, including sex, age, income level, residence, hypertension, diabetes, dyslipidemia, CCI, BMI, hemoglobin level, GFR, smoking, alcohol consumption, and regular exercise status. However, for the METS-IR, the model was adjusted for the aforementioned factors except for the BMI. Hazard ratios (HRs) and their corresponding 95% confidence intervals (CIs) were calculated to determine the association between the surrogate markers of IR and CRC. The proportional hazard assumption was evaluated using the Schoenfeld residuals test with the logarithm of the cumulative hazard function based on Kaplan–Meier estimates. There was no disturbance under the assumption of a proportional hazard risk over time. Furthermore, we employed restricted cubic splines to examine HR trends, along with their 95% CIs, for CRC according to IR marker levels. We used four knots at the 5th, 35th, 65th, and 95th percentiles according to the IR marker levels, and the mean values of the IR marker levels were selected as the reference values in the restricted cubic spline. All statistical analyses were performed using SAS (version 9.4, SAS Institute Inc., Cary, NC, USA) and R 3.6.0 (R Foundation for Statistical Computing, Vienna, Austria) to ensure the accuracy and reliability of the results. Statistical significance was defined as *p* < 0.05.

### 2.5. Ethics Statement

Our research protocols were approved by an ethics committee in compliance with the Declaration of Helsinki, which outlines the ethical principles of medical research involving human subjects. Because this study fell under a certain category, formal consent was not required. The study protocol was thoroughly reviewed and approved by the Institutional Review Board of Dong-A University College of Medicine (DAUHIRB-EXP23-105).

## 3. Results

### 3.1. Baseline Characteristics

Table 1 shows the distribution of various variables, such as sex, age, income level, residence, underlying disease, BMI, blood pressure, FBG, total cholesterol, TG, HDL-C, LDL-C, hemoglobin, GFR, smoking, alcohol consumption, and regular exercise, divided into quartiles based on the TyG index. The variables were evaluated in the same manner based on the quartiles of TG/HDL-C ratio and the METS-IR. This information is presented in Supplementary Tables S2 and S3. The average value for Q4 of the TyG index was 9.4 ± 0.3, which was higher compared to the average value of 8.0 ± 0.2 for Q1. The average value for Q4 of the TG/HDL-C ratio was 5.2 ± 1.6, which was higher compared to the average value of 1.1 ± 0.2 for Q1. The average value for Q4 of the MET-IR was 42.5 ± 2.9, which was higher compared with the average value of 28.7 ± 0.1 for Q1. The Q4s of the TyG index and the TG/HDL-C ratio exhibited relatively higher BMI, blood pressure, FBG, total cholesterol, TG, LDL-C, and hemoglobin levels and proportions of smokers compared to the other groups. Conversely, the values for HDL-C and the GFR and the frequency of regular exercise were relatively lower in these groups. The METS-IR showed trends similar to those of the TyG index and TG/HDL-C ratio, but no consistent trends were observed for the proportion of smokers. These differences were statistically significant (*p* = 0.001).

### 3.2. Incidence of CRC According to the TyG Index, TG/HDL-C Ratio, and METS-IR

**Figure 2:**
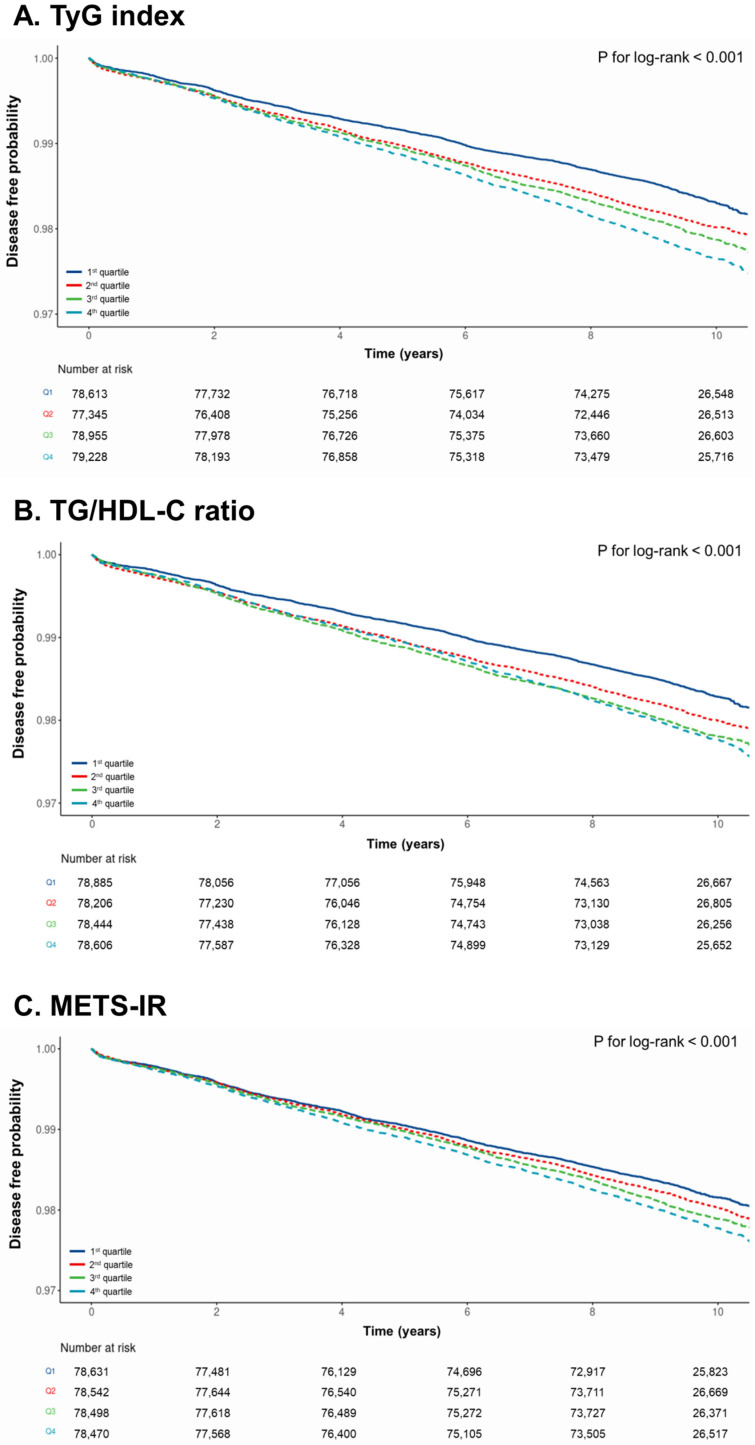
Kaplan–Meier curve for the association between surrogate markers of insulin resistance and colorectal cancer.

Figure 2 illustrates a Kaplan–Meier curve demonstrating disease-free probability across the four quartiles of the TyG index, HDL-C ratio, and METS-IR. Throughout the 10-year observation period, there was a statistically significant variance in the incidence of CRC among the groups, with a sequential increase from Q1 to Q4. All surrogate markers of IR had significantly lower disease-free probabilities from Q1 to Q4 (*p* log-rank < 0.001).

Table 2 presents the crude and adjusted HRs with 95% CIs concerning the relationship between the quartiles of the TyG index, TG/HDL-C ratio, and METS-IR and CRC risk. In the crude model, those in Q2, Q3, and Q4 had a significantly increased risk of CRC compared with individuals in Q1 of the TyG index, TG/HDL-C ratio, and METS-IR. The corresponding adjusted HRs were 1.08 (95% CI: 1.00–1.16, *p* = 0.05) and 1.10 (95% CI: 1.02–1.19, *p* = 0.01) for Q3 and Q4 of the TyG index, respectively, which were statistically significant. Similarly, risks of CRC were observed in Q3 and Q4 of the TG/HDL-C ratio, with corresponding adjusted HRs of 1.11 (95% CI: 1.03–1.20, *p* = 0.005) and 1.12 (95% CI: 1.03–1.21, *p* = 0.006), respectively, which were statistically significant. In the adjusted model, METS-IR yielded no statistical significance, with HRs of 1.05 (95% CI: 0.97–1.13, *p* = 0.24), 1.03 (95% CI: 0.95–1.11, *p* = 0.08), and 1.06 (95% CI: 0.99–1.15, *p* = 0.02) for Q2 and Q3, respectively. However, statical significance was observed with an HR of 1.10 (95% CI: 1.02–1.18, *p* = 0.02) for Q4.

**Figure 3:**
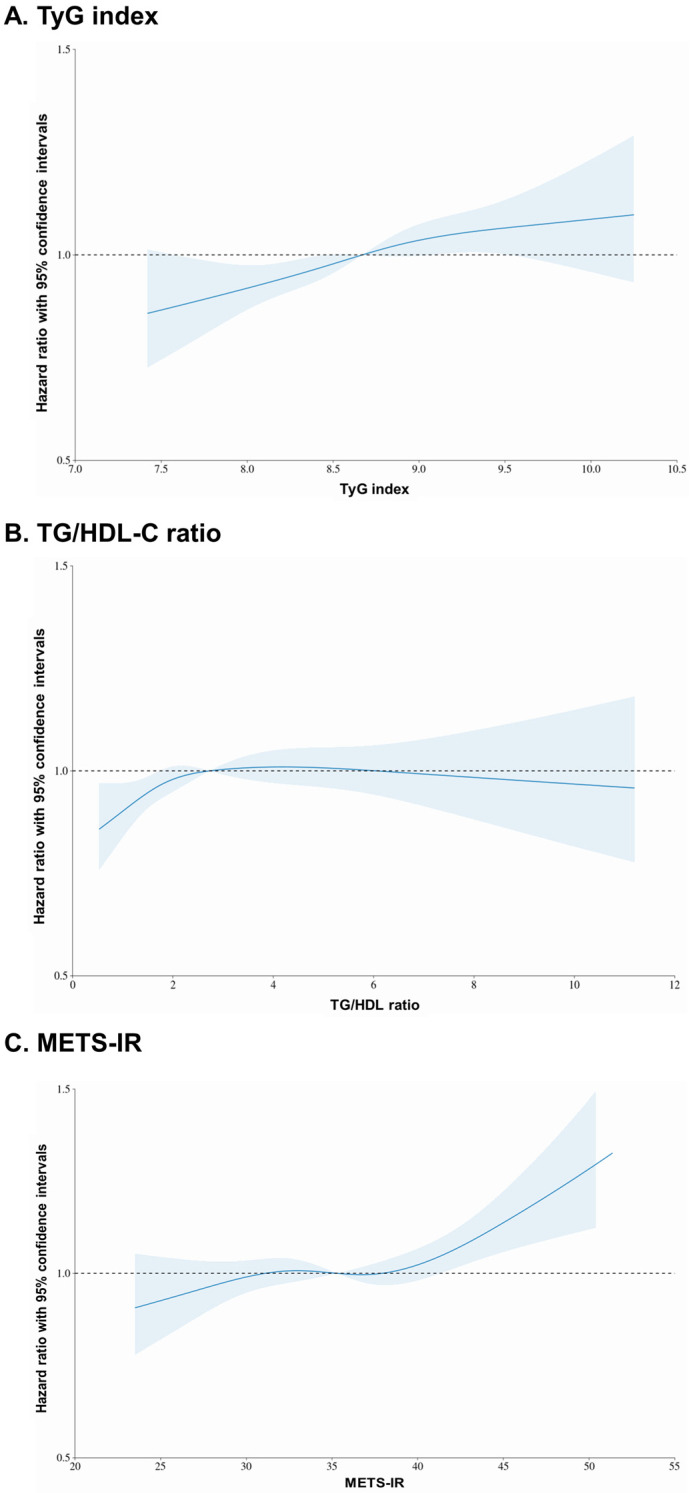
Restricted cubic spline of hazard ratio with 95% confidence intervals for colorectal cancer according to surrogate markers of insulin resistance.

Figure 3 presents a restricted cubic spline, a mathematical function employed to model nonlinear relationships between a continuous predictor variable and an outcome variable, such as the adjusted HRs with 95% CIs. The solid line represents the HR, and the blue area represents the 95% CIs. For TyG index values ≥ 8.67, the HR value increased to values > 1, and for values ≥ 9.05, all 95% CI values exceeded 1. However, for TG/HDL-C ratio values ≥ 2.73, the HR exceeded 1, but there were no values for which all CIs exceeded 1. For METS-IR values ≥ 35.3, the HR exceeded 1, and for values ≥ 41.2, all 95% CIs exceeded 1. An analysis of the overall graph using the restricted cubic spline method showed a nonlinear, positive dose–response association between the TyG index and the METS-IR in relation to the incidence of CRC, while the relationship for the TG/HDL-C ratio was less clear.

### 3.3. Subgroup Analysis

**Figure 4:**
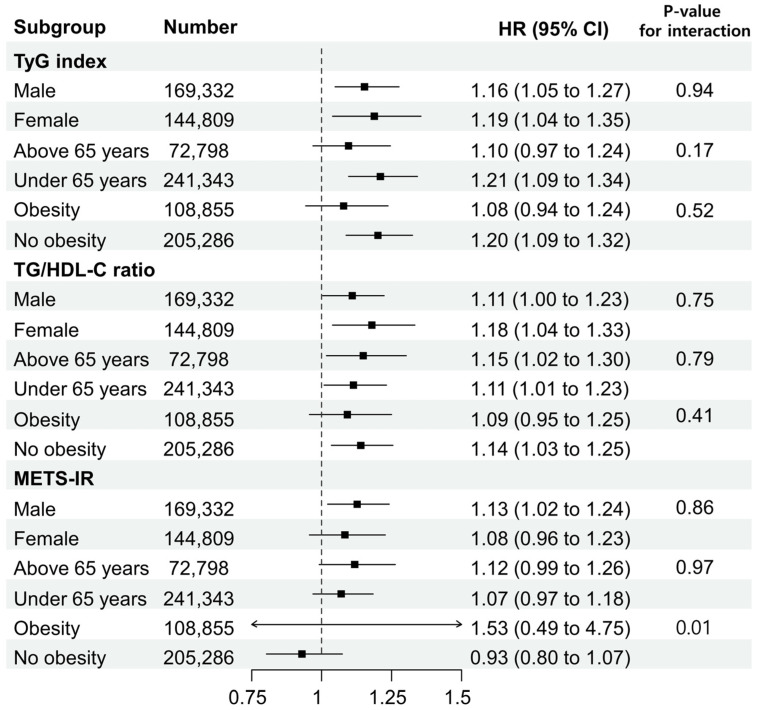
Subgroup analysis according to sex, age, and obesity status.

**Figure 5:**
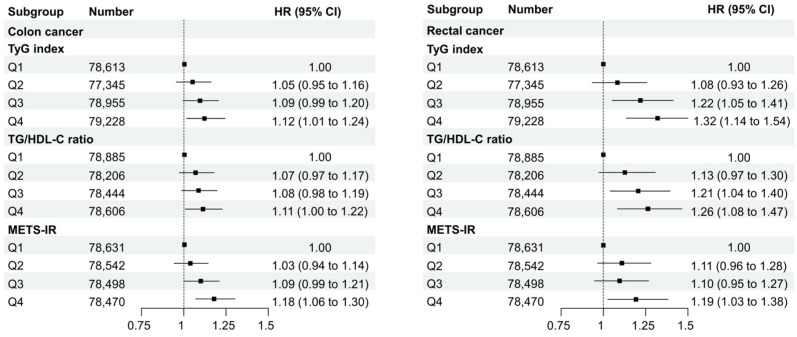
Subgroup analysis divided into colon cancer and rectal cancer.

Figure 4 and Figure 5 display the HRs of the TyG index, TG/HDL-C ratio, and METS-IR values in Q4 compared with Q1 for subgroups based on sex, age (<65 and ≥65 years), obesity status, and cancer site (colon and rectal cancers). In the TyG index and TG/HDL-C ratio, associations according to sex, age, or obesity status did not differ significantly. However, in the METS-IR, a statistically significant association was observed in the obese group compared to the non-obese group (*p*-value for interaction = 0.01). Based on the location of cancer, this study divided the cases into colon and rectal cancers and compared the TyG index, TG/HDL-C ratio, and METS-IR by quantile in both cancer types. As a result, in colon cancer, Q4 of the TyG index showed a statistically significant HR of 1.12 (95% CI: 1.01–1.24), Q4 of the TG/HDL-C ratio displayed an HR of 1.11 (95% CI: 1.00–1.22), and Q4 of METS-IR was significantly associated with an HR of 1.18 (95% CI: 1.06–1.30). In rectal cancer, Q3 and Q4 of the TyG index exhibited statistically significant HRs of 1.22 (95% CI: 1.05–1.41) and 1.32 (95% CI: 1.14–1.54), respectively. Q3 and Q4 of the TG/HDL-C ratio showed statistically significant HRs of 1.21 (95% CI: 1.04–1.40) and 1.11 (95% CI: 1.08–1.47), respectively, while the METS-IR was statistically significant only in Q4 with an HR of 1.19 (95% CI: 1.03–1.38).

## 4. Discussion

To the best of our knowledge, this large, nationwide population-based cohort study is the first conducted to concurrently evaluate the relationship between CRC and three surrogate markers of IR: the TyG index, TG/HDL-C ratio, and METS-IR. Despite variations observed across statistical techniques, this study ultimately confirmed that the TyG index is the surrogate marker for IR most strongly associated with CRC.

A consistent positive correlation between CRC and obesity, as determined by the BMI, has been observed [27,28,29,30]. The underlying mechanisms associated with obesity are likely to share similarities with those extensively recognized in the context of metabolic syndrome. Studies exploring the relationship between metabolic syndrome and the incidence of CRC have demonstrated a positive correlation [31,32,33]. Several mechanisms are involved in this process, including chronic inflammation triggered by the release of inflammatory cytokines and adipokines from adipose tissue [13,34,35]. Moreover, changes in sex hormone and adipokine levels can affect cell growth, inflammation, and other processes associated with cancer development [36,37]. Additionally, obesity and metabolic syndrome alter the gut microbiome and play a role in these mechanisms [38,39]. In addition to the aforementioned factors, IR is a key mechanism involved in this process. IR leads to an increase in hyperinsulinemia, which in turn triggers the activation of phosphoinositide 3-kinase/protein kinase B/mammalian target of rapamycin, known as the PI3K/Akt/mTOR signaling pathway in CRC [40]. IR also plays a major role in CRC development by increasing IGF-1 levels and altering PPARγ and NF-κB signaling [41,42,43].

The hyperinsulinemic–euglycemic clamp is the standard diagnostic test for IR; however, its application is limited and challenging in clinical settings. Therefore, a need for surrogate markers has emerged. The TyG index is one of these prominent markers. This marker was first proposed by Simental-Mendia et al. [16] and has been recognized as a valuable surrogate marker associated with IR in various studies, linking it to the development of type 2 diabetes and cardiovascular and cerebrovascular diseases [44,45,46]. The TG/HDL-C ratio, or METS-IR, is another, simpler marker [17,19]. Several studies have reported associations with diseases linked to IR, similar to the TyG index [20,47,48,49]. These surrogate markers of IR do not require insulin levels, which are difficult to measure in clinical settings; instead, they can be obtained using simple formulas, thus making them easy to evaluate, especially in large-scale population studies. However, despite these advantages, there is still limited research on its association with CRC; in particular, there are no studies available on the recently proposed METS-IR.

Previous studies showed a positive association between the TyG index and CRC in a study of 510,471 individuals from six European countries and a Japanese study of 27,944 individuals [23,24]. Additionally, in a study of 93,659 Chinese individuals, the TyG index and TG/HDL-C ratio were evaluated together, and both were positively associated with CRC [25]. Similar trends were observed in the present study, which is a 10-year retrospective cohort study of 314,141 Koreans. However, this study discovered notable differences from previous studies. First, this study aimed to investigate the association between three surrogate markers of IR (the TyG index, TG/HDL-C ratio, and METS-IR) and CRC, using various statistical methods. In this study, surrogate markers of IR yielded a significant association with CRC, as observed in the Kaplan–Meier curves and the Cox proportional hazards models. Moreover, a restricted cubic spline analysis revealed that the IR markers displayed a nonlinear, positive dose–response association with the incidence of CRC. Although there was variation across statistical techniques, this study confirmed that the TyG index, TG/HDL-C ratio, and METS-IR are surrogate markers for IR that are strongly associated with CRC.

Second, this study used a large dataset to conduct subgroup analyses that included factors such as sex, age, and obesity status. Additionally, analyses were performed based on the location of the cancer. In the subgroup analyses of the TyG index and TG/HDL-C ratio, which were found to be statistically significant in the adjusted model of the Cox proportional hazard analysis, there were no differences between the groups in their associations with CRC based on sex, age, or obesity status. In the subgroup analyses of the METS-IR, statistical significance was observed between the obese and non-obese groups, while in the other groups excluding this, there was no statistical significance, similar to other markers.

Although there is some variability among studies, previous studies investigating the relationship between CRC and obesity or metabolic syndrome indicated weaker associations in females compared with males [50,51]. The observed sex differences could be attributed to variations in the prevalence and age of onset of metabolic syndrome between sexes or potentially influenced by the protective effects of estrogen, which may induce apoptosis and inhibit cell proliferation [52]. However, the TyG index, TG/HDL-C ratio, and METS-IR evaluated in this study demonstrated no significant differences in their association with CRC between males and females. Considering prior research indicating that higher amounts of visceral and hepatic adipose tissue may lead to increased IR in men compared with women and the absence of protective effects from estrogen [53], it is essential to interpret the findings of this subgroup analysis with caution. Further research is required to understand this relationship better.

This study is associated with some limitations. First, it employed a retrospective study design that utilized only measurements of TG, FBG, HDL-C, and BMI at the time of the health examination; thus, it did not consider any changes in these values during the 10-year follow-up period. Consequently, the potential impact of fluctuations in these indicators on their association with CRC could not be assessed. Second, because the data were collected from a large-scale screening program in Korea, the generalizability of the findings to other ethnic groups is limited. Third, there may be hidden covariates that cannot be extracted from the NHIS-HealS database. Factors that may influence the development of CRC include a family history of CRC, a history of colorectal polyp resection, inflammatory bowel disease, a sedentary lifestyle, an inadequate intake of fruits and vegetables, a low-fiber and high-fat diet, and excessive consumption of processed meat. However, these factors were not considered in this study.

Despite these limitations, to our knowledge, this study was the first to compare three surrogate markers of IR, namely the TyG index, TG/HDL-C ratio, and METS-IR, with CRC. Moreover, the study benefitted from a large cohort with nationwide population-based data from the NHIS-HealS database, which was standardized to ensure the reliability of the results. In addition, we employed a diverse set of statistical techniques to explore the relationship between IR markers and CRC.

## 5. Conclusions

In conclusion, the TyG index, TG/HDL-C ratio, and METS-IR were positively correlated with CRC incidence in Koreans.

## Figures and Tables

**Table 1:** Baseline characteristics of the study population according to the TyG index.

All Subjects (*n* = 314,141)	TyG Index				*p*-Value
	1st Quartile, Q1 (*n* = 78,613)	2nd Quartile, Q2 (*n* = 77,345)	3rd Quartile, Q3 (*n* = 78,955)	4th Quartile, Q4 (*n* = 79,228)	
Demographics					
Age (years)	57.8 (8.5)	59.0 (8.8)	59.4 (8.8)	59.1 (8.7)	<0.001
Sex (%)					<0.001
Male	35,857 (45.6)	39,296 (50.8)	43,783 (55.5)	50,396 (63.6)	
Female	42,756 (54.4)	38,049 (49.2)	35,172 (44.5)	28,832 (36.4)	
Income level (%)					<0.001
1st quartile	11,242 (14.3)	10,919 (14.1)	10,853 (13.7)	10,911 (13.8)	
2nd quartile	16,832 (21.4)	16,019 (20.7)	15,876 (20.1)	16,087 (20.3)	
3rd quartile	22,312 (28.4)	22,529 (29.1)	23,464 (29.7)	24,290 (30.7)	
4th quartile	28,227 (35.9)	27,878 (36.0)	28,762 (36.4)	27,940 (35.3)	
Residence (%)					<0.001
Urban	51,988 (66.1)	50,093 (64.8)	50,240 (63.6)	50,027 (63.1)	
Rural	26,625 (33.9)	27,252 (35.2)	28,715 (36.4)	29,201 (36.9)	
Underlying disease					
Hypertension (%)	28,077 (35.7)	34,020 (44.0)	39,418 (49.9)	44,785 (56.5)	<0.001
Diabetes (%)	3707 (4.7)	6449 (8.3)	10,498 (13.3)	22,659 (28.6)	<0.001
Dyslipidemia (%)	18,337 (23.3)	26,124 (33.8)	34,566 (43.8)	44,624 (56.3)	<0.001
Charlson comorbidity index					<0.001
0	40,254 (51.2)	36,737 (47.5)	35,754 (45.3)	32,843 (41.5)	
1	21,967 (27.9)	21,470 (27.8)	21,568 (27.3)	21,147 (26.7)	
2	9434 (12.0)	10,521 (13.6)	11,105 (14.1)	11,800 (14.9)	
≥3	6958 (8.9)	8617 (11.1)	10,528 (13.3)	13,438 (17.0)	
Health screening					
Body mass index (kg/m^2^)	23.1 (2.6)	23.8 (2.7)	24.3 (2.7)	24.8 (2.6)	<0.001
Systolic blood pressure (mmHg)	121.6 (14.8)	124.5 (15.0)	126.4 (15.0)	128.6 (15.1)	<0.001
Diastolic blood pressure (mmHg)	75.4 (9.7)	77.1 (9.8)	78.2 (9.7)	79.6 (9.9)	<0.001
Fasting blood glucose (mg/dL)	90.9 (11.4)	95.8 (13.9)	100.5 (17.7)	114.2 (32.9)	<0.001
Total cholesterol (mg/dL)	189.9 (34.0)	198.2 (35.4)	203.6 (36.9)	209.5 (39.2)	<0.001
Triglyceride (mg/dL)	67.7 (14.4)	101.9 (16.3)	140.3 (24.9)	224.8 (67.4)	<0.001
HDL cholesterol (mg/dL)	58.7 (12.9)	55.4 (12.6)	52.2 (12.1)	49.0 (15.3)	<0.001
LDL cholesterol (mg/dL)	117.4 (33.3)	122.4 (35.9)	123.3 (36.2)	116.3 (40.3)	<0.001
Hemoglobin (g/dL)	13.5 (1.4)	13.7 (1.5)	13.9 (1.5)	14.2 (1.5)	<0.001
Glomerular filtration rate (mL/min/1.73 m^2^)	80.3 (31.4)	78.7 (28.6)	78.0 (31.5)	77.3 (31.9)	<0.001
Current smoker (%)	9172 (11.7)	11,452 (14.8)	13,996 (17.7)	18,125 (22.9)	<0.001
Alcohol consumption (%)	27,427 (34.9)	28,735 (37.2)	31,686 (40.1)	37,154 (46.9)	<0.001
Regular exercise (%)	4156 (5.3)	3683 (4.8)	3521 (4.5)	3207 (4.0)	<0.001
TyG index	8.0 ± 0.2	8.5 ± 0.1	8.8 ± 0.1	9.4 ± 0.3	<0.001

**Table 2:** Hazard ratios and 95% confidence intervals for the incidence of colorectal cancer according to the TyG index, TG/HDL ratio, and METS-IR.

Subjects (n = 314,141)	Events	Follow-Up Duration (Person-Years)	Incidence Rate (per 1000 Person-Years)	Hazard Ratios (95% Confidence Intervals)			
				Crude	*p*-Value	Adjusted *	*p*-Value
TyG index							
Q1 (*n* = 78,613)	1266	746,824	1.70	1.00 (reference)		1.00 (reference)	
Q2 (*n* = 77,345)	1468	727,043	2.02	1.18 (1.10–1.27)	<0.001	1.08 (1.00–1.16)	0.05
Q3 (*n* = 78,955)	1606	742,177	2.16	1.27 (1.18–1.37)	<0.001	1.10 (1.02–1.19)	0.01
Q4 (*n* = 79,228)	1772	744,743	2.38	1.40 (1.31–1.51)	<0.001	1.16 (1.07–1.25)	<0.001
TG/HDL-C ratio							
Q1 (*n* = 78,885)	1287	749,408	1.72	1.00 (reference)		1.00 (reference)	
Q2 (*n* = 78,206)	1497	735,136	2.04	1.18 (1.09–1.27)	<0.001	1.07 (0.99–1.15)	0.09
Q3 (*n* = 78,444)	1642	737,374	2.23	1.29 (1.20–1.39)	<0.001	1.11 (1.03–1.20)	0.005
Q4 (*n* = 78,606)	1686	738,896	2.28	1.32 (1.23–1.42)	<0.001	1.12 (1.03–1.21)	0.006
METS-IR ^†^							
Q1 (*n* = 78,631)	1367	739,131	1.85	1.00 (reference)		1.00 (reference)	
Q2 (*n* = 78,542)	1485	746,149	1.99	1.08 (1.00–1.16)	0.04	1.05 (0.97–1.13)	0.24
Q3 (*n* = 78,498)	1588	745,731	2.13	1.15 (1.07–1.24)	<0.001	1.07 (0.99–1.15)	0.07
Q4 (*n* = 78,470)	1672	745,465	2.24	1.22 (1.13–1.31)	<0.001	1.10 (1.02–1.18)	0.02

* The model was adjusted for age, sex, income level, residence, hypertension, diabetes, dyslipidemia, Charlson comorbidity index, body mass index, hemoglobin level, glomerular filtration rate, smoking, alcohol consumption, and regular exercise status. ^†^ In the METS-IR, the model was adjusted as for the aforementioned model except for the body mass index.

## Data Availability

Data are contained within the article and Supplementary Materials.

## Supplementary Materials

The following supporting information can be downloaded at https://www.mdpi.com/article/10.3390/jcm13061628/s1, Table S1. Definitions for clinical variables; Table S2. Baseline characteristics of study population according to the TG/HDL-C ratio; Table S3. Baseline characteristics of study population according to the METS-IR.
