# Supplementary material for: Association between Surrogate Markers of Insulin Resistance and the Incidence of Colorectal Cancer in Korea: A Nationwide Population-Based Study
Source: J Clin Med. 2024 Mar 12;13(6):1628. doi: 10.3390/jcm13061628 (PMC10971512; doi:10.3390/jcm13061628)
Supplement: Supplementary file 1 [file jcm-13-01628-s001.zip › jcm-2889653-supplementary.pdf]

Supplementary Materials

Supplementary Table S1. Definitions for clinical variables.

Clinical variables	ICD-10 codes + Claim codes	Health screening
Hypertension	I10-I11 + Prescription of anti-hypertensive drugs ≥ 1	Systolic blood pressure ≥ 140 mmHg or diastolic blood pressure ≥ 90 mmHg
Diabetes	E10-E14 + Prescription of anti-diabetic drugs ≥ 1	Fasting blood glucose ≥ 126 mg/dL
Dyslipidemia	E78 + Prescription of lipid-lowering drugs ≥ 1	Total cholesterol 240 mg/dL or<br>LDL cholesterol 190 mg/dL or<br>Triglyceride 500 mg/dL or<br>HDL cholesterol < 40 mg/dL
Colorectal cancer	C18, C19, and C20	
Colon cancer	C18 and C19	
Rectal cancer	C20	

Supplementary Table S2. Baseline characteristics of study population according to the TG/HDL-C ratio.

All subjects (n = 314,141)	TG/HDL-C ratio				P-value
	1st quartile, Q1 (n = 78,885)	2nd quartile, Q2 (n = 78,206)	3rd quartile, Q3 (n = 78,444)	4th quartile, Q4 (n = 78,606)	
**Demographics**					
Age (years)	57.9 (8.4)	59.0 (8.8)	59.4 (8.9)	59.0 (8.7)	< 0.001
Sex (%)					< 0.001
Male	34,775 (44.1)	39,523 (50.5)	44,344 (56.5)	50,690 (64.5)	
Female	44,110 (55.9)	38,683 (49.5)	34,100 (43.5)	27,916 (35.5)	
Income level (%)					< 0.001
1st quartile	11,465 (14.5)	11,232 (14.4)	10,646 (13.6)	10,582 (13.5)	
2nd quartile	17,140 (21.7)	16,382 (20.9)	15,791 (20.1)	15,501 (19.7)	
3rd quartile	22,416 (28.4)	22,863 (29.2)	23,426 (29.9)	23,890 (30.4)	
4th quartile	27,864 (35.3)	27,729 (35.5)	28,581 (36.4)	28,633 (36.4)	
Residence (%)					< 0.001
Urban	52,748 (66.9)	50,434 (64.5)	49,932 (63.7)	49,234 (62.6)	
Rural	26,137 (33.1)	27,772 (35.5)	28,512 (36.3)	29,372 (37.4)	
**Underlying disease**					
Hypertension (%)	29,805 (37.8)	35,170 (45.0)	39,298 (50.1)	42,027 (53.5)	< 0.001
Diabetes (%)	7,026 (8.9)	9,549 (12.2)	12,249 (15.6)	14,489 (18.4)	< 0.001
Dyslipidemia (%)	19,288 (24.5)	25,011 (32.0)	32,370 (41.3)	46,982 (59.8)	< 0.001
Charlson comorbidity index					< 0.001

0	39,495 (50.1)	36,316 (46.4)	34,794 (44.4)	34,983 (44.5)	
1	21,815 (27.7)	21,522 (27.5)	21,486 (27.4)	21,329 (27.1)	
2	9,686 (12.3)	10,749 (13.7)	11,294 (14.4)	11,131 (14.2)	
≥3	7,889 (10.0)	9,619 (12.3)	10,870 (13.9)	11,163 (14.2)	
**Health screening**					
Body mass index (kg/m^2)	23.2 (2.7)	23.8 (2.8)	24.3 (2.7)	24.8 (2.6)	< 0.001
Systolic blood pressure (mmHg)	122.6 (15.1)	124.8 (15.2)	126.2 (15.0)	127.6 (15.0)	< 0.001
Diastolic blood pressure (mmHg)	75.9 (9.8)	77.2 (9.9)	78.1 (9.8)	79.1 (9.8)	< 0.001
Fasting blood glucose (mg/dL)	97.0 (19.7)	99.3 (22.0)	101.6 (24.0)	103.6 (23.7)	< 0.001
Total cholesterol (mg/dL)	196.2 (35.1)	198.8 (36.8)	201.7 (37.6)	204.6 (38.4)	< 0.001
Triglyceride (mg/dL)	69.7 (18.8)	102.5 (20.7)	139.4 (28.8)	224.4 (66.8)	< 0.001
HDL cholesterol (mg/dL)	64.8 (16.0)	56.0 (10.3)	50.4 (9.4)	43.9 (8.6)	< 0.001
LDL cholesterol (mg/dL)	117.6 (34.5)	122.2 (35.5)	123.4 (37.1)	116.2 (38.8)	< 0.001
Hemoglobin (g/dL)	13.5 (1.4)	13.7 (1.5)	13.9 (1.5)	14.2 (1.5)	< 0.001
Glomerular filtration rate (mL/min/1.73 m^2)	80.0 (30.8)	78.8 (28.6)	78.0 (30.4)	77.4 (33.5)	< 0.001
Current smoker (%)	8,682 (11.0)	11,513 (14.7)	14,220 (18.1)	18,330 (23.3)	< 0.001
Alcohol drink (%)	29,432 (37.3)	29,453 (37.7)	31,410 (40.0)	34,707 (44.2)	< 0.001
Regular exercise (%)	4,288 (5.4)	3,738 (4.8)	3,405 (4.3)	3,136 (4.0)	< 0.001
TG/HDL-C ratio	1.1 ± 0.2	1.8 ± 0.2	2.8 ± 0.3	5.2 ± 1.6	< 0.001

Supplementary Table S3. Baseline characteristics of study population according to the METS-IR.

All subjects (n = 314,141)	METS-IR				P-value
	1st quartile, Q1 (n = 78,631)	2nd quartile, Q2 (n = 78,542)	3rd quartile, Q3 (n = 78,498)	4th quartile, Q4 (n = 78,470)	
**Demographics**					
Age (years)	58.5 (9.2)	58.7 (8.7)	59.0 (8.6)	59.0 (8.5)	< 0.001
Sex (%)					< 0.001
Male	35,344 (44.9)	40,347 (51.4)	45,285 (57.7)	48,356 (61.6)	
Female	43,287 (55.1)	38,195 (48.6)	33,213 (42.3)	30,114 (38.4)	
Income level (%)					< 0.001
1st quartile	11,699 (14.9)	11,028 (14.0)	10,652 (13.6)	10,546 (13.4)	
2nd quartile	17,649 (22.4)	16,433 (20.9)	15,404 (19.6)	15,328 (19.5)	
3rd quartile	22,516 (28.6)	22,980 (29.3)	23,177 (29.5)	23,922 (30.5)	
4th quartile	26,767 (34.0)	28,101 (35.8)	29,265 (37.3)	28,674 (36.5)	
Residence (%)					< 0.001
Urban	51,570 (65.6)	50,958 (64.9)	50,522 (64.4)	49,298 (62.8)	
Rural	27,061 (34.4)	27,584 (35.1)	27,976 (35.6)	29,172 (37.2)	
**Underlying disease**					
Hypertension (%)	25,650 (32.6)	33,413 (42.5)	39,813 (50.7)	47,424 (60.4)	< 0.001
Diabetes (%)	4,724 (6.0)	8,081 (10.3)	12,144 (15.5)	18,364 (23.4)	< 0.001
Dyslipidemia (%)	18,877 (24.0)	25,919 (33.0)	33,077 (42.1)	45,778 (58.3)	< 0.001
Charlson comorbidity index					< 0.001

0	41,071 (52.2)	37,421 (47.6)	35,262 (44.9)	31,834 (40.6)	
1	21,331 (27.1)	21,851 (27.8)	21,672 (27.6)	21,298 (27.1)	
2	9,363 (11.9)	10,464 (13.3)	11,032 (14.1)	12,001 (15.3)	
≥3	6,866 (8.7)	8,806 (11.2)	10,532 (13.4)	13,337 (17.0)	
**Health screening**					
Body mass index (kg/m^2)	21.0 (1.5)	23.3 (1.4)	24.8 (1.5)	27.0 (2.1)	< 0.001
Systolic blood pressure (mmHg)	121.3 (15.3)	124.4 (15.0)	126.6 (14.8)	128.8 (14.7)	< 0.001
Diastolic blood pressure (mmHg)	75.2 (9.8)	77.0 (9.8)	78.4 (9.7)	79.7 (9.7)	< 0.001
Fasting blood glucose (mg/dL)	94.0 (15.8)	97.8 (19.3)	101.8 (22.5)	107.9 (28.2)	< 0.001
Total cholesterol (mg/dL)	199.1 (35.5)	200.9 (36.9)	201.2 (37.8)	200.1 (38.2)	< 0.001
Triglyceride (mg/dL)	94.0 (41.4)	117.2 (52.8)	142.6 (65.1)	182.3 (80.0)	< 0.001
HDL cholesterol (mg/dL)	63.5 (16.2)	55.8 (11.0)	50.8 (10.1)	45.0 (9.4)	< 0.001
LDL cholesterol (mg/dL)	117.0 (35.2)	121.6 (35.6)	122.0 (37.7)	118.8 (37.6)	< 0.001
Hemoglobin (g/dL)	13.4 (1.4)	13.7 (1.4)	14.0 (1.5)	14.2 (1.5)	< 0.001
Glomerular filtration rate (mL/min/1.73 m^2)	80.3 (29.4)	79.1 (31.0)	77.8 (30.4)	76.9 (32.7)	< 0.001
Current smoker (%)	12,839 (16.3)	12,173 (15.5)	13,129 (16.7)	14,604 (18.6)	< 0.001
Alcohol drink (%)	28,572 (36.3)	30,875 (39.3)	32,656 (41.6)	32,899 (41.9)	< 0.001
Regular exercise (%)	3,571 (4.5)	3,846 (4.9)	3,698 (4.7)	3,452 (4.4)	< 0.001
METS-IR	28.7 ± 1.9	33.2 ± 1.0	36.9 ± 1.1	42.5 ± 2.9	< 0.001
